# Improvement in Surface Hardness and Wear Resistance of ADI via Arc-Deposited CrAlSiN Multilayer Films

**DOI:** 10.3390/ma18092107

**Published:** 2025-05-04

**Authors:** Cheng-Hsun Hsu, Hong-Wei Chen, Chun-Yin Lin, Zhe-Hong Chang

**Affiliations:** Department of Mechanical and Materials Engineering, Tatung University, Taipei City 10451, Taiwan; tonychen318@gmail.com (H.-W.C.); rupert475@gmail.com (C.-Y.L.); world0147max@gmail.com (Z.-H.C.)

**Keywords:** austempered ductile iron, cathodic arc deposition, CrN/Al(Si)N multilayer film, surface hardness, wear resistance

## Abstract

In this study, as-cast ductile iron was austempered to produce austempered ductile iron (ADI). A CrAlSiN film was then deposited on the surface of ADI specimens using the cathodic arc deposition (CAD) method. The gas flow ratio of Ar/N_2_ varied (2, 2.5, and 3) under different processing parameters, designated as S1, S2, and S3, respectively. The composition, structure, hardness, adhesion, and wear resistance of the coated specimens were analyzed to evaluate the effect of the gas flow ratio on surface hardness and abrasion resistance. The experimental results indicated that CrN/Al(Si)N nano-multilayered films were successfully synthesized using oppositely positioned dual targets (Cr and AlSi) reacting with N_2_ gas during the CAD process. The coatings significantly enhanced the surface hardness and wear resistance of ADI. A comparison of the three coating conditions with varying gas flow ratios revealed that as the Ar/N_2_ gas flow ratio decreased (i.e., N_2_ gas flow increased), the surface hardness of the coated ADI specimens increased while the abrasion rate decreased. Among the tested conditions, S1 exhibited the highest hardness (1479 HV_0.1_) and the lowest wear rate (1.6 × 10^−^⁶ g/m).

## 1. Introduction

Ductile iron is a kind of ferrous material that was developed around 1960. It has been widely used for engineering applications in recent decades due to its excellent mechanical properties, such as hardness, strength, and ductility [1]. Austempered ductile iron (ADI) is produced by subjecting ductile iron to an austempering heat treatment, which enhances its mechanical properties. The heat treatment process involves three main steps: first, the ductile iron is heated to the austenitizing temperature (~950 °C) to achieve complete austenitization. It is then rapidly quenched to the austempering temperature (~350 °C) and subsequently air-cooled to room temperature. This process results in a microstructure composed of acicular ferrite and high-carbon retained austenite, which contribute to the improved properties of ADI [2,3,4]. Due to its special microstructure, ADI is almost twice as strong as the regular ASTM grades of ductile iron while maintaining high elongation and toughness characteristics. In addition, ADI has good fatigue and wear resistance and is, therefore, commonly used in mechanical products that require high contact stress, such as gear components. Various surface modification techniques are commonly used to extend the service life of metallic components. Enhancing the surface hardness and wear resistance of ADI is expected to further improve its durability and applicability [5,6]. However, for ADI, the temperature used in surface modification must be kept below its austempering temperature, as excessive heat can degrade its microstructure and compromise its mechanical properties.

Physical vapor deposition (PVD) is a thin-film technology in which material is physically transformed from a solid or liquid source into a vapor and then deposited onto the surface of a target substrate, forming a thin film. Unlike chemical vapor deposition (CVD), PVD mainly relies on physical processes rather than chemical reactions. It is well known that the process temperature of PVD is generally lower than that of other surface modification methods, such as CVD, carburizing, and nitriding [7,8,9]. In recent years, PVD technology has been widely employed to deposit nitride films on metallic components, enhancing their surface properties. Cathodic arc deposition (CAD) is a popular PVD method that operates at 200–300 °C for its processing temperature. Some studies have used CAD technology to coat ADI substrates; the results showed that the coating temperature almost had no influence on ADI’s microstructure [10,11]. Thus, CAD is a suitable surface modification method for ADI. Regarding coating types, extensive research has explored various nitride coatings, including binary [12,13,14,15,16,17,18,19,20], ternary [21,22,23,24,25], and multi-element coatings [26,27,28,29,30], as well as modifications in film-layer numbers [31,32,33,34,35]. Different compositions and layer configurations result in distinct coating characteristics, offering a range of performance benefits. For instance, Maskavizan et al. showed that the CrN coatings enhanced the corrosion resistance of steel in chloride and acidic media [12]. Yang et al. pointed out that CrAlN coatings had good adhesion to nitrided steel substrates and improved hardness [21]. In a case study of four-elemental coatings, Teles et al. found that CrAlSiN-PVD coatings had good wear resistance, which indicated that any related research would be interesting [29]. Lin et al. reported that CrN/AlN multilayers had good resistance to oxidation at high temperatures [31]. In addition, a study was also conducted to apply (TiCrCuAlSi)N multi-element coatings on the H13 tool steel by varying the gas flow ratio of nitrogen to argon using the CAD method. The results revealed that the coatings exhibited excellent wear resistance and were influenced by the gas flow ratio [33]. These examples collectively demonstrate that nitride coatings containing Cr, Al, Si, and N elements display outstanding performance characteristics, particularly in multilayer structures that enhance the benefits of the film. However, research on the application of these coatings to ADI material remains limited.

Therefore, this study utilized CAD coating technology to deposit CrAlSiN multilayers on ADI substrates while varying the Ar/N_2_ gas flow ratio. The effects of these coatings on the surface hardness and abrasion resistance of ADI were then analyzed. The findings from this study could provide valuable insights into ADI’s engineering applications.

## 2. Materials and Methods

### 2.1. ADI Material Preparation

**Figure 1:**
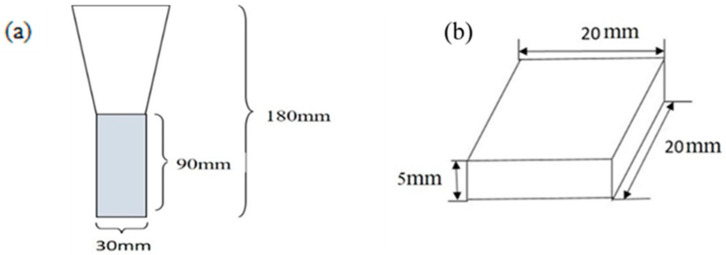
(**a**) Diagram of Y-block casting and (**b**) dimensions of the specimen in this study.

The raw material used in this study was FCD450 ductile iron produced by Y-type casting. Its appearance and dimensions are shown in Figure 1a. The material was sourced from Tatung Company, Taiwan. To avoid the location in the casting where shrinkage holes might easily occur, only the lower part of the casting was sampled and machined into the 20 × 20 × 5 mm^3^ specimens (shown in Figure 1b). The chemical composition of the iron used is listed in Table 1.

**Figure 2:**
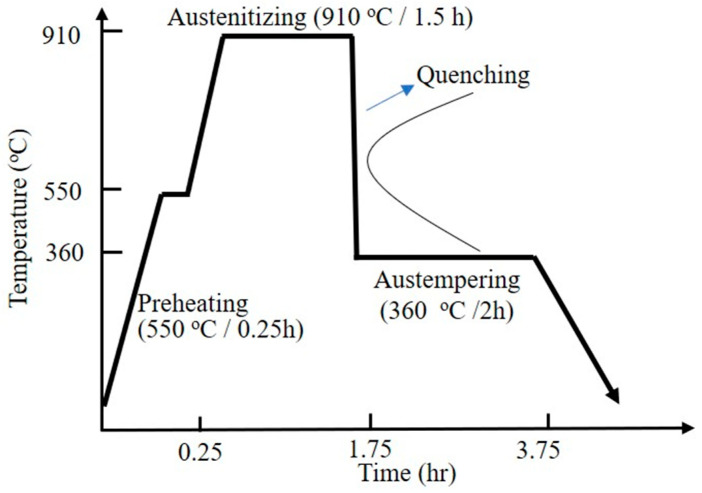
Schematic diagram of austempering treatment in this study.

The above specimens were then austempered to obtain ADI microstructure. The heat treatment procedure is shown in Figure 2 and briefly described as follows.

Building on the experience of previous studies [10,11], the specimens were first preheated in a 550 °C salt bath furnace for 0.25 h. They were then transferred to another furnace at 910 °C for 1.5 h to achieve a fully austenitized matrix. Next, the specimens were quenched into a 360 °C salt bath furnace for 2 h to undergo austempering isothermal treatment. Finally, they were removed from the furnace and air-cooled to room temperature, forming the ADI.

### 2.2. CAD Coating Treatment

**Figure 3:**
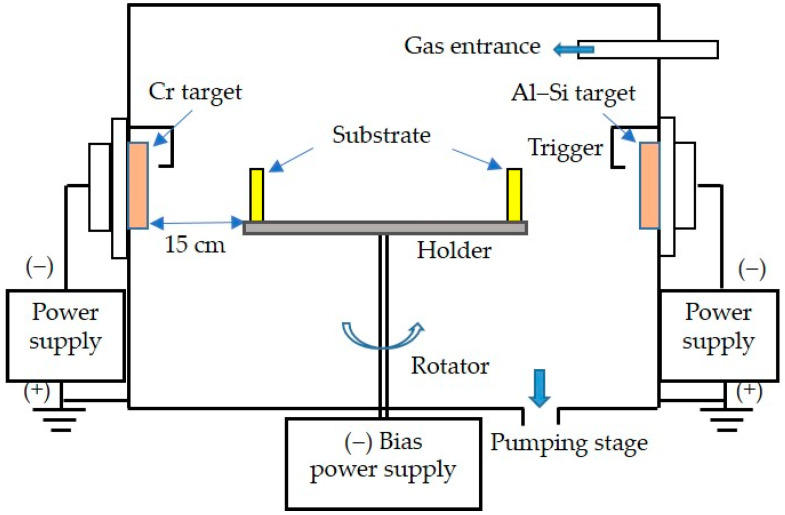
Schematic diagram of the CAD system used in this study.

The coating treatment was carried out in this study using a CAD system. The schematic diagram of the coating equipment is shown in Figure 3. Based on the available CAD devices and the results of previous studies [32,33], the proper parameters for this coating experiment were set as follows. Two targets, Cr99.5wt.% and Al88wt.%-Si12wt.%, were used and placed in opposite positions of the vacuum furnace. The bias value was set to −100 V, the target current was set to 60 A, and the two gases containing argon (Ar) and nitrogen (N_2_) were fed into the vacuum furnace. Predictably, the CrN/AlSiN multilayered coatings could be obtained by varying the gas flow ratios of Ar/N_2_ to 2, 2.5, and 3, numbered S1, S2, and S3, respectively. In other words, the Ar/N_2_ ratio was increased by reducing the N_2_ flux at a fixed total gas flow rate. The main parameters of the deposition process for the experiment are listed in Table 2.

### 2.3. Characterization and Observation of Coatings

In this study, the characterization of the obtained coatings is described below. (1) The composition of the films was analyzed using a field emission electron probe microanalyzer (EPMA, JEOL JXA-8530F Plus, Tokyo, Japan). EPMA analysis was conducted using an accelerating voltage of 15 kV, a beam current of 20 nA, and a focused beam size of approximately 1 µm. For nitrogen quantification, calibration was carried out using a hexagonal boron nitride (h-BN) standard, which was selected for its stable composition and well-characterized nitrogen content. (2) The structure of the coatings was analyzed using a multi-function X-ray diffractometer (D8 Discover X-ray Diffraction System, Bruker, Karlsruhe, Germany), where the films were analyzed at a low angle of incidence (1°) with a scanning range of 20–80°. The JCPDS (Joint Committee on Powder Diffraction Standards) card was used to identify the crystallographic phases of the films from the X-ray diffraction patterns. (3) A field emission scanning electron microscope (FESEM, HITACHI SU-8000, Ibaraki, Japan) was used to measure the coating thickness and observe the surface morphology of the films. Additionally, FESEM was further used to observe multilayer morphology in the film. (4) The surface roughness (Ra value) of the uncoated and coated specimens was measured using a surface roughness tester (Mitutoyo Surf test SV-400, Kanagawa, Japan). Five Ra values were averaged for each specimen. (5) The adhesion strength quality (ASQ) of the coatings was evaluated using Rockwell-C indentation testing with a load of 150 Kg. The damage to the coatings was compared with a defined ASQ basis, where the strengths of HF1-HF4 were acceptable for adhesion, and HF5-HF6 represented insufficient adhesion (HF is the German short form for adhesion strength) [36].

### 2.4. Hardness Measurements

**Figure 4:**
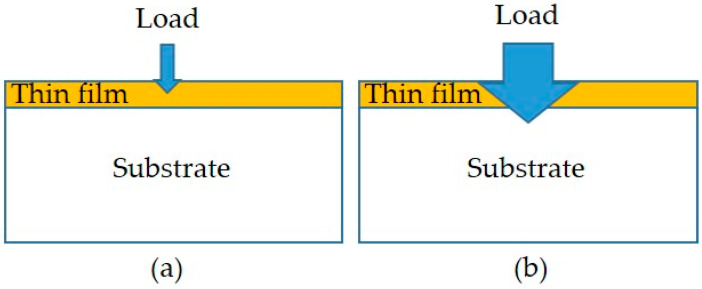
Schematic diagrams of the hardness tests: (**a**) nanoindentation test and (**b**) microhardness test.

In this study, two types of hardness testing methods were conducted: the nanoindentation test without penetrating the film and the microhardness test with a penetrated film. Schematic diagrams of the two hardness tests are shown in Figure 4. The details are given below.

To analyze the hardness of the films, nanoindentation measurements were performed using a nanoindenter (Hysitron TI980 TriboIndenter, Minneapolis, MN, USA) with a diamond Berkovich tip. According to the Oliver and McHargue method [37], both the values of hardness (H) and elastic modulus (E) for the films were measured by analyzing force–displacement curves under a force of 5 μN. The averages of 5 indentations for each film were reported with the standard deviation of both the H and E values.

On the other hand, a micro-Vickers hardness tester with a 100 g load was used to measure the microhardness of the specimens. Since the indentation depth penetrated through the thin film into the substrate for the coated specimen, the measured hardness value affected by the substrate was defined as the surface hardness. This is different from previously mentioned values for film hardness. Measurements were taken at five random locations on each specimen and averaged to represent the surface hardness of the coated specimens.

### 2.5. Wear Test

Based on the previous wear testing experiences [32,33], the study performed wear tests using a ball-on-disk tribometer (CSM Instruments, Peseux, Switzerland). The testing conditions were as follows: (1) no lubricant; (2) a circular track with a 5 mm diameter against a 6 mm diameter WC-6%Co ball; (3) a wear speed of 10 cm/s; (4) a load of 3 N; (5) a 25 °C ambient temperature; and (6) 65% relative humidity. The relationship between the friction coefficient and the total travel distance of 500 m was continuously recorded during the testing. The wear rate was derived from the specimen’s weight loss before and after the wear test, which was evaluated using a microbalance with an accuracy of 1 × 10^−4^ g. Furthermore, the worn surface of the specimens was observed using the FESEM.

## 3. Results and Discussion

### 3.1. Analysis of Coating Composition and Structure

**Figure 5:**
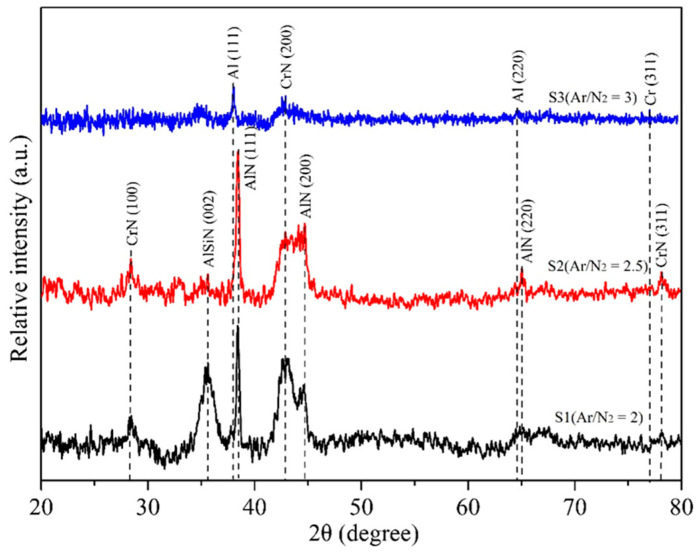
XRD patterns of the coatings in this study.

The compositions of the three films obtained were analyzed using EPMA, as listed in Table 3. Additionally, their coating structures were examined by XRD, as shown in Figure 5. The data in Table 3 indicate that as the Ar/N_2_ flow ratio increased from two to three, the nitrogen content in the coating decreased, while the Cr, Al, and Si contents increased. This trend occurs because a higher Ar/N_2_ flow ratio (i.e., a reduced N_2_ gas flow) lowers the probability of Al and Cr reacting with nitrogen ions, leading to more unreacted metal particles being directly deposited into the film. Furthermore, the Si content in the coatings was relatively low, resulting from the lower Si concentration of the Al-Si (88:12) target used in this study.

Figure 5 presents the XRD patterns of the film structures for the three coated specimens. The S1 specimen exhibited characteristic CrN peaks corresponding to the (111), (200), and (311) crystal planes at 2θ = 37.5°, 43.2°, and 76.2°, respectively, along with AlN characteristic peaks of (111), (200), and (220) crystal planes at 2θ = 38.4°, 44.6°, and 65.2°. These results indicate that the S1 film primarily consisted of CrN and AlN phases. Moreover, a small amount of AlN doped with Si, forming Al(Si)N, was also detected in the film. Such a result implied that a trace of the Al(Si)N phase was mixed in the AlN composition. For the case of S2, the CrN and AlN peaks in the XRD pattern were slightly weaker than those in S1 due to the lower nitrogen content (see Table 3). The XRD pattern of S3 further revealed that, with the lowest nitrogen content, the characteristic peaks of nitrides became less prominent, except for a small CrN(200) peak. Moreover, the trace amounts of Al and Cr peaks were observed in S3, while no Si peak was detected. This suggests that the Al and Cr elements remained in a crystalline state due to their higher concentrations in the film, whereas Si likely existed in an amorphous form. From the analytical results of the three coatings, it was found that nitrogen content significantly influenced the composition of the CrAlSiN coating structure. As the Ar/N_2_ ratio increased (i.e., the nitrogen content decreased), the CrN and AlN phase content reduced, leading to the deposition of some Cr and Al metal elements within the film. The formation of CrN and AlN phases during the PVD process is closely related to the availability of nitrogen in the plasma atmosphere. Nitrogen acts as a reactive gas that chemically bonds with sputtered metal atoms (Cr and Al) to form their respective nitrides. When nitrogen partial pressure is sufficient, it promotes the formation of stoichiometric nitride phases such as CrN and AlN, resulting in a well-defined crystalline structure. However, as the nitrogen content in the plasma decreases (i.e., with an increased Ar/N_2_ ratio), the reduced nitrogen availability limits the formation of these nitrides. As a result, a portion of the sputtered Cr and Al atoms remains unreacted and may be deposited in their metallic or sub-stoichiometric nitride forms. This incomplete nitridation can disrupt the crystallinity of the coating and lead to the presence of amorphous regions or less-defined phases, as reflected by the less pronounced XRD peaks in the low-nitrogen specimen (e.g., S3). This phenomenon has also been reported in some studies on PVD processes [38,39].

### 3.2. Morphology Observation and Adhesion of Coatings

**Figure 6:**
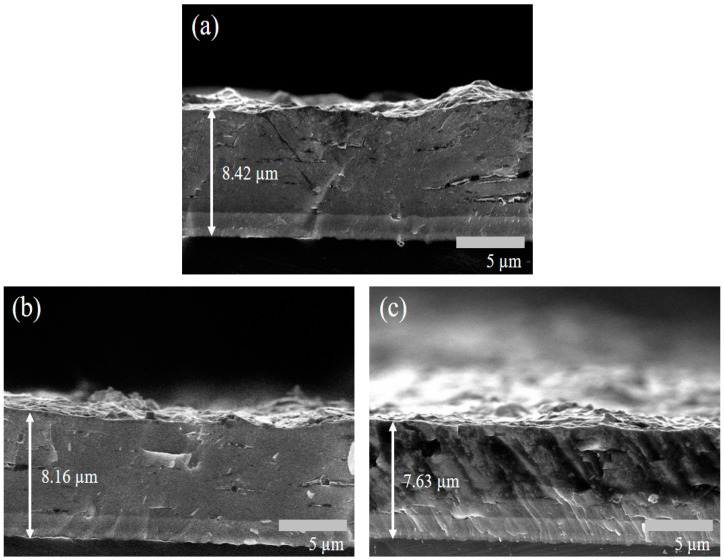
SEM cross-sectional view of the coatings: (**a**) S1, (**b**) S2, and (**c**) S3.

Cross-sectional observations of the coated specimens using FESEM were conducted to examine the effect of the Ar/N_2_ flow ratio on film thickness. As shown in Figure 6, film thickness decreased with an increasing Ar/N_2_ flow ratio, with average thickness values in the following order: S1 (8.42 µm) > S2 (8.16 µm) > S3 (7.63 µm). This indicates that a higher Ar/N_2_ flow ratio results in a slight reduction in film thickness. This is because argon (Ar) serves as an inert gas primarily responsible for sustaining plasma and generating sputtering ions through collisions in the PVD process. In contrast, nitrogen (N_2_) acts as a reactive gas that binds with sputtered metal atoms to form nitrides. When the Ar/N_2_ ratio increases (i.e., the nitrogen content decreases), the reduced availability of nitrogen limits nitride formation, which leads to a decrease in the overall deposition rate.

**Figure 7:**
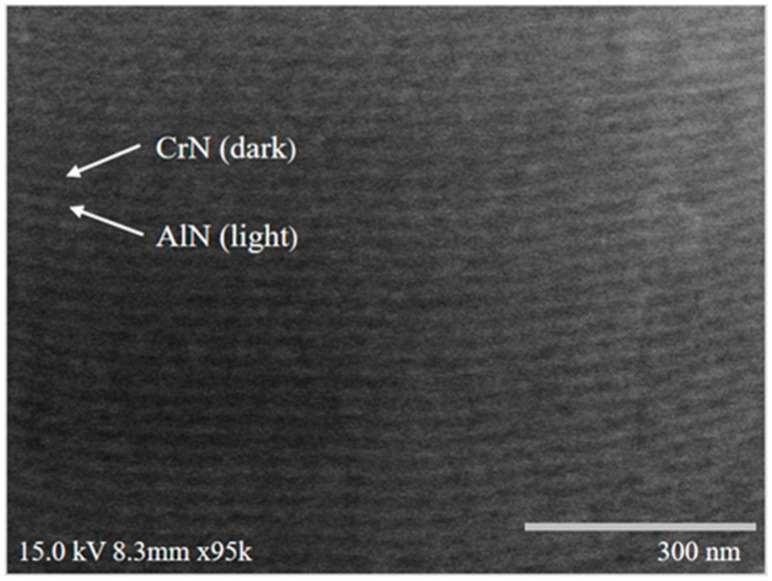
Cross-sectional view of the S1 multilayered film using SEM at high magnification.

**Figure 8:**
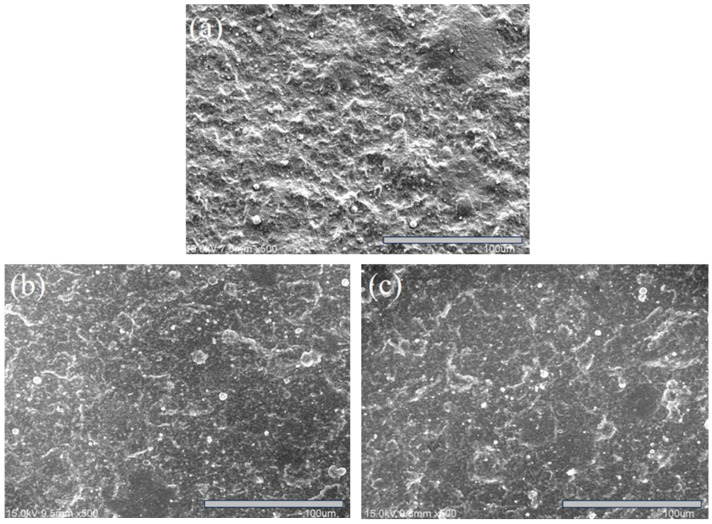
SEM surface morphologies of the coated specimens: (**a**) S1, (**b**) S2, and (**c**) S3.

It is also observed that S3 appears to have smaller grain sizes compared to S1 and S2 (Figure 6c vs. Figure 6a,b), resulting in a thinner and more compact film under the same deposition time. This microstructural difference may also explain the less pronounced XRD peaks observed for S3 (Figure 5). The further high-magnification FESEM analysis of the S1 film cross-section revealed a well-defined multilayer morphology, as expected from the experimental design (Figure 7). This multilayered structure consists of alternating CrN and AlN layers, with CrN appearing as dark regions (~14 nm thick) and AlN as light regions (~17 nm thick). Figure 8 presents the SEM images of the surface morphology of the three coated specimens. The images reveal the presence of microdroplets on the CAD-coated surfaces, which is a well-known drawback of the CAD process that affects surface roughness [40,41]. Among the specimens, S1 (Figure 8a) exhibits the highest number of microdroplets, while S2 and S3 (Figure 8b,c) show a relatively lower droplet density.

**Figure 9:**
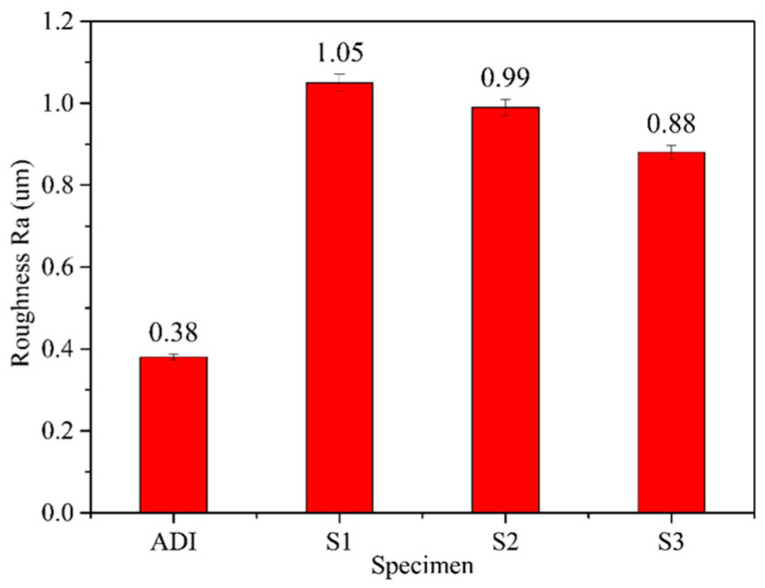
A comparison of surface roughness for ADI and the coated specimens.

To further assess surface roughness, the Ra values of the coated specimens were compared, as shown in Figure 9. The data indicate that S1 has the highest surface roughness (Ra = 1.05 µm), followed by S2 (Ra = 0.99 µm), with S3 having the lowest roughness (Ra = 0.88 µm). This trend suggests that surface roughness slightly decreases as the Ar/N_2_ flow ratio increases. Moreover, the Ra values of all three coated specimens were higher than that of the uncoated one (Ra = 0.38 µm). Thus, the results of the surface roughness measurement are consistent with the SEM observation of the surface morphology in Figure 8.

**Figure 10:**
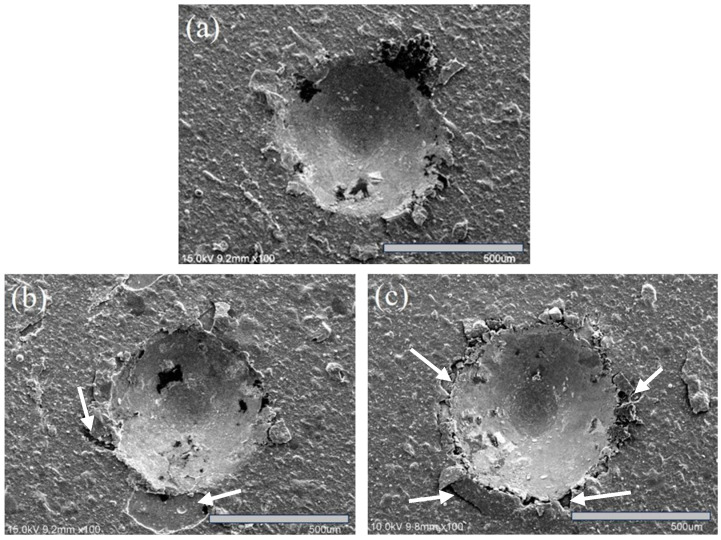
Fractured morphology of the coated specimens from the Rockwell-C adhesion test: (**a**) S1, (**b**) S2, and (**c**) S3. The arrows indicate cracked and flaked coatings.

For the evaluation of coating adhesion, the VDI-3198 specification [36] was adopted, and the adhesion strength quality (ASQ) was assessed based on specification criteria. Figure 10 presents the SEM images of the three coated specimens after indentation testing. In Figure 10a, the S1 specimen shows no significant cracks or flaking around the indentation, corresponding to an HF1 rating. In Figure 10b, the S2 specimen exhibits some flaking around the indentation, classifying it as HF2. Meanwhile, Figure 10c shows that the S3 specimen has noticeable cracks and flaking, resulting in an HF3 rating. Despite these differences, the adhesion of all three coatings meets the acceptable requirements outlined in the VDI-3198 specification.

### 3.3. Analysis of Coating Hardness

**Figure 11:**
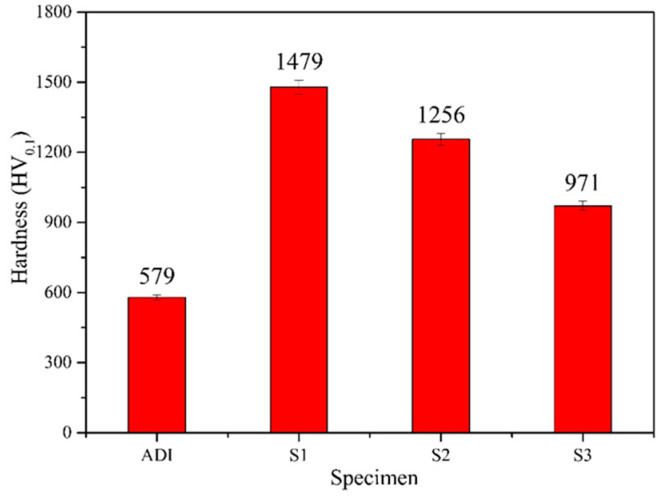
A comparison of surface hardness for ADI and the coated specimens.

In this study, the Vickers hardness tester was used to analyze surface hardness. As shown in Figure 11, all three coated specimens exhibited a significant increase in surface hardness compared to the uncoated ADI substrate. The hardness values corresponded to the following order: S1 (1479 HV) > S2 (1256 HV) > S3 (971 HV) > ADI (579 HV), with S1 demonstrating the highest microhardness. The S1-coated specimen could increase the surface hardness value of the ADI substrate up to 2.5 times. Therefore, S1 provided the most significant enhancement for the surface hardness of ADI.

On the other hand, the hardness testing of the films was conducted using a nanoindentation analyzer. Since the indentation depth did not reach the substrate, the measurements of hardness (H) and elastic modulus (E) were obtained without influence from the substrate effect. These data and the H/E values calculated are presented in Table 4. The data in Table 4 indicate that the highest hardness (H) and elastic modulus (E) were observed in the S1 specimen, followed by S2 and then S3. This suggests a trend of increasing H and E values with a higher Ar/N_2_ flow ratio. The XRD analysis supports this result, as the presence of CrN and AlN nitrides in the films contributed to this trend (see Figure 4). Specifically, a higher nitrogen gas flow leads to the formation of a more stable nitride layer, which enhances the hardness and elastic modulus. The hardness of the three films followed the order S1 (13.3 GPa) > S2 (11.6 GPa) > S3 (8.5 GPa), which aligned with the surface hardness trend. This indicated a positive correlation between film hardness and surface hardness in the coated specimens. In other words, a higher film hardness resulted in a higher surface hardness of the coated specimen.

Consequently, the S1 specimen contained the highest concentration of nitrides, whereas S3 had more layers composed of Al and Cr metal elements due to the reduced nitrogen gas flow, leading to a lower hardness value. Additionally, the ratio of film hardness to elastic modulus (H/E) affects the wear resistance of the coated material [37], as discussed in the next section.

### 3.4. Analysis of Wear Behavior

**Figure 12:**
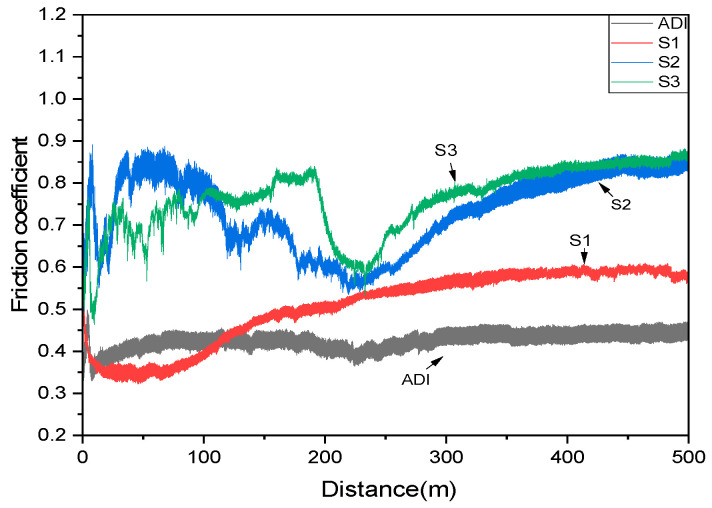
The comparison of friction coefficients for ADI and the coated specimens.

**Figure 13:**
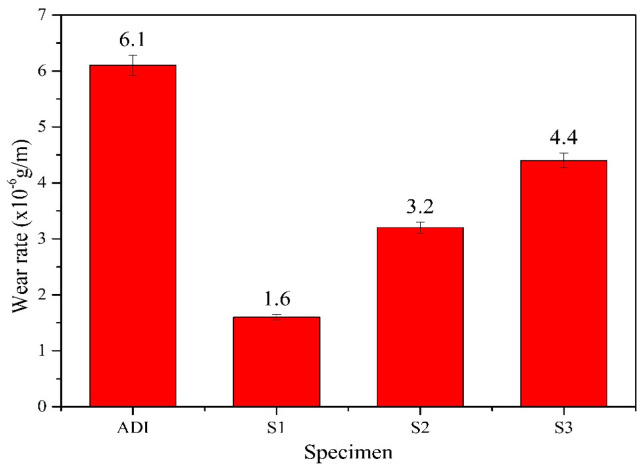
The comparison of wear rates for ADI and the coated specimens.

**Figure 14:**
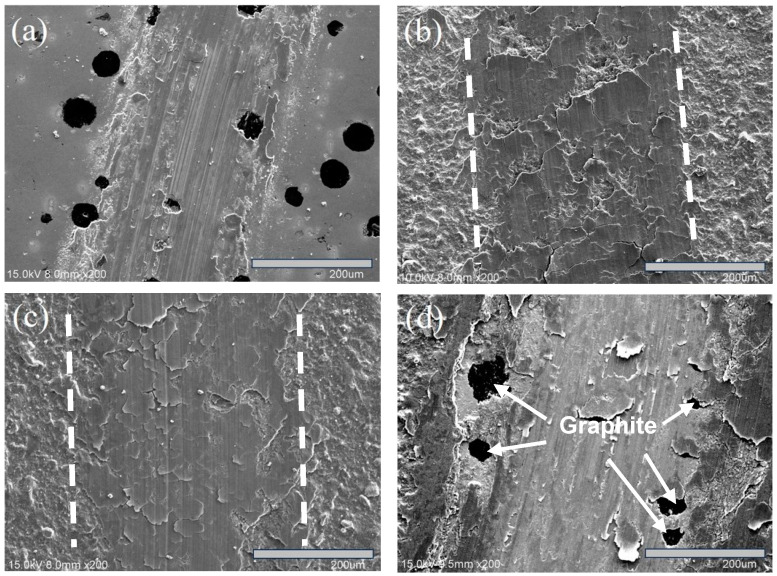
The surface wear tracks of the specimens after wear tests: (**a**) ADI, (**b**) S1, (**c**) S2, and (**d**) S3. The arrows indicate exposed graphite.

The relationship between the friction coefficient and wear distance for each specimen after wear testing is presented in Figure 12. Figure 13 compares the wear rates of the specimens. Additionally, SEM analysis was conducted to examine the wear traces on the specimen surfaces, as shown in Figure 14.

Figure 12 shows that the coated specimens had higher friction coefficients than the uncoated ADI. This is primarily due to the microdroplets formed during the coating process, which increased surface roughness. In contrast, the uncoated ADI contained graphite on its surface to provide a lubricating effect, resulting in the lowest friction coefficient of 0.45. Among the coated specimens, S1 had a slightly higher surface roughness than S2 and S3 but exhibited a lower friction coefficient of approximately 0.55. In comparison, S2 and S3 had similar friction coefficients of around 0.85. It can be inferred that the friction coefficient of the coated specimens is influenced not only by surface morphology but also by the coating structure. As previously mentioned, the S1 structure contained a higher concentration of stabilized CrN and AlN hard ceramic phases, leading to a lower coefficient of friction. In contrast, S2 and S3 exhibited a less stable coating structure, resulting in a higher friction coefficient. These differences in friction behavior are expected to influence the wear performance of the specimens.

From the comparison of the wear rate of each specimen in Figure 13, it can be seen that all the coated specimens have lower wear rates (1.6–4.4 × 10^−6^ g/m) than the uncoated ADI (6.1 × 10^−6^ g/m), so the wear resistance of ADI can be significantly improved by the CrAlSiN multilayer coatings. The wear rate of the three coated specimens was further compared, and the wear rate of S1 was the lowest (1.6 × 10^−6^ g/m), followed by S2 (3.2 × 10^−6^ g/m), and then S3 (4.4 × 10^−6^ g/m). Such a result is consistent with the aforementioned trend of H/E values, i.e., the larger the H/E value for the coating material, the smaller the wear rate. The findings can also be supported by some related literature [33,37,42].

Figure 14 presents the SEM images of the wear tracks for each specimen after wear testing, showing a correlation with the wear rate. As seen in Figure 14a, the uncoated specimen exhibited the highest wear rate, with deep scratches forming around graphite. Among the coated specimens, Figure 14b,c show that S1 and S2 retained most of their coating layers, though some minor peeling was observed. In contrast, Figure 14d reveals that S3 experienced severe cracking and flaking, exposing nodular graphite in the substrate, which contributed to its lower wear resistance. Additionally, the measured wear track widths followed the corresponding trend: S1 (325.7 µm) < S2 (362.9 µm) < S3 (485.7 µm), confirming that S1 exhibited the best wear resistance.

## 4. Conclusions

This study provides a significant contribution to surface engineering and materials science by successfully depositing CrAlSiN nano-multilayer coatings onto ADI substrates and investigating the influence of varying Ar/N_2_ flow ratios on the film composition, structure, and performance.

1. Deposition and composition control: The study successfully deposited CrAlSiN nano-multilayer films with adjustable composition by varying the Ar/N_2_ flow ratios. As the Ar/N_2_ ratio increased, the nitrogen content decreased, while the Cr and Al content increased, along with a slight increase in Si. This ability to control the film’s composition made it easier to design coatings with specific hardness and wear-resistant properties.
2. Structural insights: The coatings had a nano-multilayer structure mainly composed of CrN and AlN phases. As the nitrogen content decreased with higher Ar/N_2_ ratios, metallic Cr and Al emerged within the coatings. These structural transitions directly impacted the coating properties and provided valuable insights into the interplay between the process parameters and phase evolution in nitride coatings.
3. Adhesion performance: The coatings deposited at Ar/N_2_ ratios of two to three displayed excellent adhesion to the ADI substrate (HF1–HF3), with the best performance observed at a ratio of two (HF1). This suggests that optimized gas flow ratios not only influence the film composition but also contribute to strong interfacial bonding, which is crucial for coating durability in demanding applications.
4. Hardness enhancement: The CrAlSiN coatings substantially improved the surface hardness of ADI, increasing it from 579 HV to a maximum of 1479 HV. This nearly 2.5-fold enhancement in hardness, particularly at an Ar/N_2_ ratio of two, underscores the coating’s potential to significantly extend the functional lifespan of the ADI components.
5. Wear resistance improvement: The coatings also yielded a marked improvement in wear resistance, with the lowest wear rate (1.6×10^−^⁶ g/m) recorded at the optimal Ar/N_2_ ratio. These results not only highlight the effectiveness of the CrAlSiN coating in reducing material loss during frictional contact but also offer practical guidance for industrial applications where wear resistance is paramount.

## Figures and Tables

**Table 1:** Chemical composition of the experimental iron used in this study (wt.%).

C	Si	Mn	P	S	Mg	Fe
3.48	2.83	0.25	0.06	0.04	0.04	Bal.

**Table 2:** The CAD processing parameters of CrAlSiN coatings used in this study.

Parameter	Value
Two targets	Cr (99.5%) and Al-Si (88:12%)
Working pressure (Torr)	2 × 10^−2^
Gas flow ratio (Ar/N_2_)	2 (S1), 2.5 (S2), and 3 (S3)
Cathode current (A)	60
Substrate bias (V)	–100
Ar^+^ Bombardment (V)	–700
Substrate temperature (°C)	250
Rotation speed of carrier (rpm)	4
Distance between target and substrate (mm)	150
Deposition time (min)	55

**Table 3:** Chemical composition of the films analyzed by EPMA (at.%).

Specimen	Cr	Al	Si	N
S1	23.4	26.5	2.8	47.3
S2	24.5	27.7	3.1	44.7
S3	25.8	29.7	3.6	40.9

**Table 4:** Hardness (H), elastic modulus (E), and H/E values of the films in this study.

Specimen	Hardness (GPa)	Elastic Modulus (GPa)	H/E
S1	13.3 ± 0.3	206 ± 3	0.064 ± 0.004
S2	11.6 ± 0.4	189 ± 4	0.061 ± 0.002
S3	8.5 ± 0.6	163 ± 6	0.052 ± 0.006

## Data Availability

The original contributions are presented in this study’s article; further inquiries can be directed to the corresponding author.
